# Photocatalytic hydrogenation of nitrobenzene to aniline over titanium(iv) oxide using various saccharides instead of hydrogen gas†

**DOI:** 10.1039/d1ra05953j

**Published:** 2021-09-30

**Authors:** Kazuya Imamura, Kazuma Ikeuchi, Yuki Sakamoto, Yushiro Aono, Takahiro Oto, Ayumu Onda

**Affiliations:** Department of Chemistry and Biotechnology, Research Laboratory of Hydrothermal Chemistry, Faculty of Science and Technology, Kochi University 2-5-1 Akebono-cho Kochi 780-8520 Japan imamura-kazuya@kochi-u.ac.jp

## Abstract

Bare TiO_2_ photocatalyst almost quantitatively converted nitrobenzene to aniline with various saccharides without the use of hydrogen gas. Although aniline was formed when any saccharide was used, the use of disaccharides (lactose, maltose, and sucrose) decreased the reaction rate. The rate of photocatalytic hydrogenation of nitrobenzene using saccharides is determined by the degradation rate of saccharides at positive holes. When glucose was used, formic acid, arabinose, glyceraldehyde and lactic acid were obtained, which are products that are consistent with the product of the photocatalytic oxidation of glucose.

## Abstract 2

10 kinds of saccharides were investigated as hydrogen source in photocatalytic hydrogenation of nitrobenzene to aniline.

## Graphical abstract

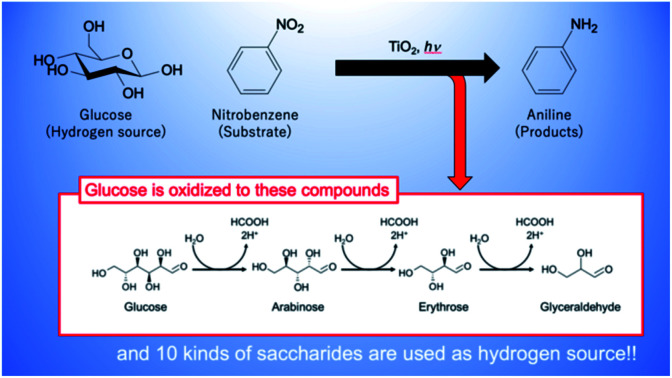

## Introduction

Hydrogenation is one of the most important processes in the chemical industry and is indispensable for the production of various chemical products. Among them, hydrogenation of nitro aromatics to amino aromatics is a very useful reaction because amino aromatics are key intermediates for the manufacture of pharmaceuticals, agrochemicals, plastics, dyes and pigments.^1^ Generally, hydrogenation of nitro aromatics to amino aromatics is achieved by high-pressure hydrogen at a high temperature. Therefore, the development of a method with mild conditions for production of amino aromatics is desired. In addition, production of dihydrogen (H_2_) requires tremendous energy. Natural gas steam reforming is the most common method for industrial H_2_ production, providing about 90% of the H_2_ in the world.^2^ For large-scale production, H_2_ is produced by steam reforming of natural gas, methane, over metal catalysts such as nickel (Ni) under high pressure (20–40 bar) and at a high temperature (500–800 °C). However, the steam reforming method has two problems: methane, an exhaustible resource, is used as the H_2_ source and the reaction conditions are very severe. Biomass, obtained from plants, is an abundantly available renewable energy source and is perceived as a carbon neutral source of energy.^3^ Moreover, environmental issues due to the disposal of agricultural waste can be efficiently managed through the utilization of biomass.^4^ Thus, biomass is a promising feedstock for H_2_ production.^5^ Many researchers have studied reforming of biomass by thermochemical gasification, pyrolysis and anaerobic digestion.^6^ The steam reforming process of bio-oil, which is produced from biomass pyrolysis, has also been studied.^3^ However, these processes require a large amount of energy and tar that deactivates the catalyst is generated.

A photocatalytic reaction is a redox process caused by photoirradiation. When a photocatalyst is irradiated by light, electrons are excited to the conduction band, leaving positive holes in the valence band. The thus-formed electrons and holes cause reduction and oxidation, respectively. A photocatalytic reaction proceeds under room temperature and atmospheric pressure. Therefore, photocatalytic reforming of biomass is one of the promising methods for H_2_ production. A pioneering study on photocatalytic H_2_ production from biomass was conducted in 1980 by Kawai and Sakata.^7^ They investigated H_2_ production from several type of biomass, including cellulose, starch, dead insects and waste materials, using a photocatalytic process.^8^ Their works showed the feasibility of producing H_2_ from biomass by a photocatalytic process, and photocatalytic processes for H_2_ production gained considerable attention.^9^ Although both thermal H_2_ production and photocatalytic H_2_ production from biomass are good approaches for obtaining H_2_, storage and transport H_2_ are difficult because H_2_ should be liquefied under high pressure and cooling. Therefore, hydrogenation of nitro aromatics using biomass as a H_2_ source is a very attractive reaction.^10^

Saccharides are abundant and renewable resources that are contained in plants. Glucose obtained from depolymerization of cellulose, a main component of lignocellulosic biomass,^11^ is an abundant and renewable resource.^12^ Therefore, many researchers have studied utilization of glucose including H_2_ production from glucose by various routes such as photocatalytic processes,^7^ catalytic reforming^1*e*^ and enzymatic procedures.^13^ However, there have been few reports of a photocatalytic hydrogenation reaction using glucose. In this study, photocatalytic hydrogenation of nitro aromatics over TiO_2_ using various types of saccharides instead of H_2_ was investigated.

## Experimental

All reagents with guaranteed reagent (FUJIFILM Wako Chemicals) grades were used without further purification.

### 1. Preparation of metal-loaded TiO_2_ by the photodeposition method

By using the photodeposition method, various metals were loaded on TiO_2_ (JRC-TIO-15, P 25) as co-catalysts. The metal sources were hydrogen hexachloroplatinate(iv) (H_2_PtCl_6_, 98.5%), palladium(ii) acetate ((CH_3_COO)_2_Pd, 97.0%), hydrogen tetrachloroaurate(iii) tetrahydrate (HAuCl_4_·4H_2_O, 99%), silver nitrate (AgNO_3_, 99.8%), copper(ii) sulfate pentahydrate (CuSO_4_·5H_2_O, 99.5%) and rhodium(iii) chloride trihydrate (RhCl_3_, 99.5%). Bare TiO_2_ powder was suspended in a 2-propanol solution (5 cm^3^) containing a metal source in a test tube. The test tube was sealed with a rubber septum under argon (Ar). After stirring for 0.5 h in the dark, a 400 W high-pressure mercury arc (KOIKE PRECISION INSTRUMENTS, Hyogo, Japan) was used as the source of UV light to reduce the metal source to a metallic state on the surface of TiO_2_. Photodeposition was performed for 1 h at 298 K in a water bath. The metal-loaded TiO_2_ was washed with distilled water and then dried for 1 day under vacuum.

### 2. Photocatalytic hydrogenation of nitrobenzene in aqueous suspensions of TiO_2_ under irradiation of UV light

In a typical run, TiO_2_ powder (50 mg, JRC-TIO-15) was suspended in a distilled water (5 cm^3^) containing nitrobenzene (50 μmol) and saccharides (2000 ppm) in a test tube. The tube was sealed with a rubber septum under Ar and then photoirradiated at a wavelength >300 nm by a high-pressure mercury arc (400 W, KOIKE PRECISION INSTRUMENTS, Hyogo, Japan) with magnetic stirring at 298 K. After the reaction, the gas phase was analyzed by a gas chromatograph (GC-8A, Shimadzu, Kyoto) equipped with Prapak-QS columns (GL Sciences Inc., Tokyo). After the reaction, the TiO_2_ photocatalyst was removed by filtration, and then the amounts of nitrobenzene and aniline, hydrogenated products, were determined by high-performance liquid chromatography (Variable Wavelength UV Monitor Detector and 655 Liquid Chromatograph pump, Hitachi, Tokyo) with a TSKgel ODS-100Z column (5 μm, Tosoh, Tokyo) at room temperature (eluent: water : acetonitrile = 30 : 70).

### 3. Adsorption of nitrobenzene and aniline on TiO_2_ in the presence of glucose

Typically, a TiO_2_ sample (50 mg) was suspended in 5 cm^3^ of 2000 ppm aqueous glucose solution containing nitrobenzene (50 μmol) or aniline (50 μmol) in a test tube, and the test tube was sealed with a rubber septum under Ar. When effect of saccharides and co-catalyst were investigated, 1000 ppm of glucose was used, respectively. The test tube was magnetically stirred for 24 h in the dark. After the suspension had been filtered to remove particles, the amounts of nitrobenzene and aniline, hydrogenated products, were determined by high-performance liquid chromatography (Variable Wavelength UV Monitor Detector and 655 Liquid Chromatograph pump, Hitachi, Tokyo) with a TSKgel ODS-100Z column (5 μm, Tosoh, Tokyo) at room temperature (eluent: water : acetonitrile = 30 : 70). The amounts of oxidation products of glucose were determined by high-performance liquid chromatography (RID-6A Detector, Shimadzu, Kyoto and Shodex DS-4 pump, Showa Denko, Tokyo) with a shodex sugar SH1821 column at 50 °C (eluent: 5 mM sulfonic acid aqueous solution).

## Results and discussion

Fig. S1† shows the effects of different co-catalysts on the photocatalytic hydrogenation of nitrobenzene in aqueous suspensions of metal-loaded TiO_2_ in the presence of glucose. Generally, effective charge separation of excited electrons and holes is achieved by loading of a metal co-catalyst on a TiO_2_ photocatalyst. However, bare TiO_2_ showed the highest activity in the present system. Shiraishi *et al.* reported that oxygen vacancies work as adsorption sites for oxygen atoms of a nitro group in photocatalytic hydrogenation of nitrobenzene.^14^ In the photocatalytic hydrogenation of nitrobenzene using saccharides, the adsorption sites may be coated by the metal co-catalysts. When metals with lower hydrogen overvoltage was used, photocatalytic hydrogenation of nitrobenzene would compete with H_2_ evolution. On the other hand, Tada *et al.* reported that silver (Ag) works efficiently in photocatalytic hydrogenation of nitrobenzene in an alcoholic suspension of TiO_2_.^15^ When Ag–TiO_2_ is used in photocatalytic hydrogenation of nitrobenzene using glucose, unknown by-products were detected by HPLC. These results indicate that Ag may catalyze side reactions such as condensation of intermediates for aniline formation and converted glucose, *e.g.*, formic acid, arabinose, glyceraldehyde, and lactic acid.

Table 1 shows the effects of saccharides on aniline yield in photocatalytic hydrogenation of nitrobenzene in aqueous suspensions of bare TiO_2_. Although aniline was formed when any saccharides were used, the use of disaccharides (lactose, maltose, and sucrose) decreased the reaction rate. Thakur *et al.* reported that the reaction rates of photocatalytic degradation of disaccharides are lower than the rate of degradation of monosaccharides.^16^ These results indicate that the rate of photocatalytic hydrogenation of nitrobenzene using saccharides is determined by the degradation rate of saccharides at positive holes.

**Table 1:** Effects of different kinds of saccharides on yield of aniline in photocatalytic hydrogenation of nitrobenzene (50 μmol) in the presence of glucose (1000 ppm, 28 μmol) after 60 min photoirradiation

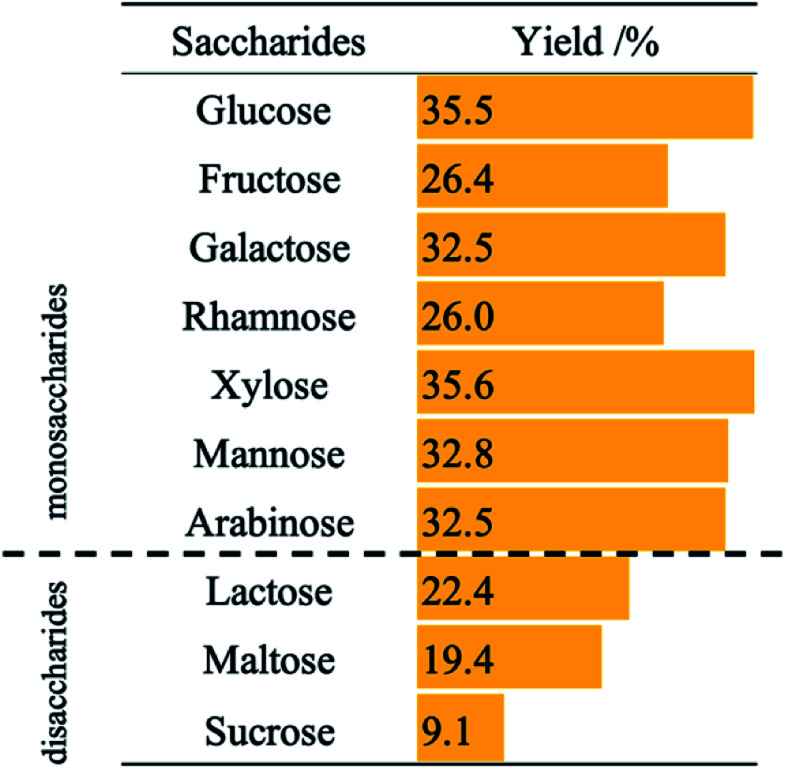

**Scheme 1:**
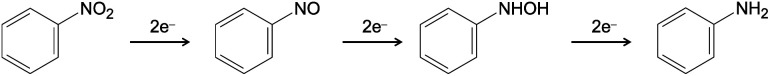
Intermediates in reduction of nitrobenzene to aniline.

Fig. S2† shows the effect of glucose concentration on yield of aniline after 1.5 h photoirradiation. Aniline yield after 1 h irradiation was increased with increase in the concentration of glucose under 2000 ppm. Since photocatalytic hydrogenation of nitrobenzene to aniline needs 6e^−^ and 6H^+^ electrochemically (Scheme 1), the concentration of H^+^ may be important for photocatalytic reduction (hydrogenation). Actually, Chen *et al.* reported that the rate of photocatalytic reduction (hydrogenation) of nitrobenzene to aniline was increased in an acidic solution because the TiO_2_ surface, on which the isoelectric point is 6.8, is positively charged, and then negatively charged oxygen atoms in the nitro group are adsorbed on the TiO_2_ surface electrostatically.^17^

Fig. S3† shows the yields of aniline after 1 h photoirradiation in acidic (0.1 M HCl) and basic (0.1 M NaOH) solutions. Unexpectedly, the highest yield of aniline was obtained in a neutral solution, while an acidic or basic condition drastically decreased the generation of aniline. Sanwald *et al.* investigated H_2_ evolution from saccharides over Rh–TiO_2_ and found that lower pH decreased the rate of H_2_ formation because hydrogenolysis of aldoses was inhibited.^18^ That is the reason why the reaction rate of photocatalytic hydrogenation of nitrobenzene was decreased in an acidic solution. In a basic solution, the TiO_2_ surface is negatively charged, decreasing adsorption of negatively charged nitro groups. In addition, protons (H^+^) as the hydrogen source are also decreased. Therefore, photocatalytic hydrogenation of a nitro group was inhibited in a basic solution. Therefore, the reaction rate of photocatalytic hydrogenation of nitrobenzene is highest in a neutral solution, which is also favourable from the point of view of reaction rate and green chemistry.

**Fig. 1:**
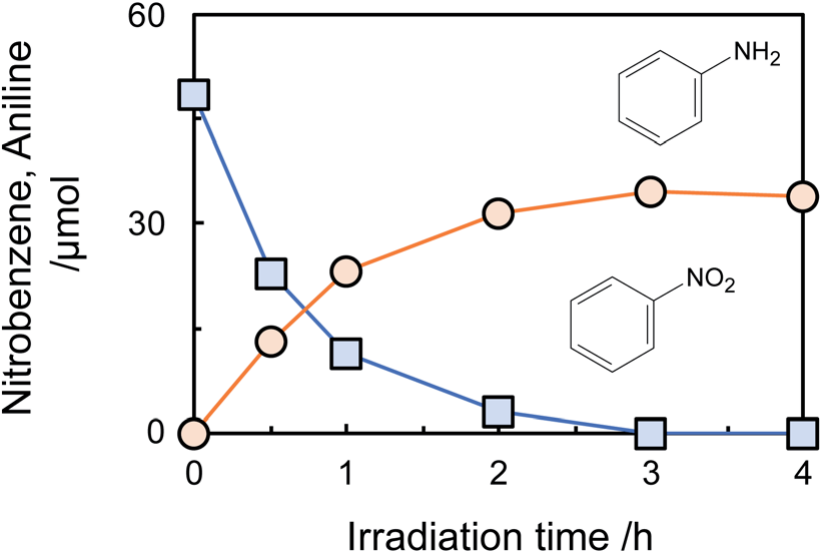
Time courses of the amount of nitrobenzene consumed and aniline formed in an aqueous suspension of TiO_2_ containing 2000 ppm (56 μmol) of glucose under an Ar condition.

Fig. 1 shows time courses of the amount of nitrobenzene consumed and aniline formed in photocatalytic hydrogenation of nitrobenzene to aniline in an aqueous suspension (neutral pH) of bare TiO_2_ in the presence of glucose (2000 ppm) under a deaerated condition. Nitrobenzene was decreased with photoirradiation and was completely consumed after 3 h, while aniline was formed in 81% yield calculated by following equation.
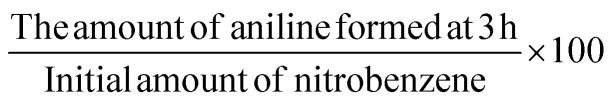


Aniline was not decreased by further photoirradiation, indicating that reoxidation of aniline at positive holes did not occur. To reveal the stability of aniline against photocatalytic reaction in the present system, photocatalytic reaction of aniline in an aqueous suspension of TiO_2_ in the presence of 2000 ppm of glucose was investigated (Fig. S4†). The amount of aniline was almost constant for 2 h photoirradiation, showing that a high yield of aniline was achieved because aniline formed in the photocatalytic hydrogenation of nitrobenzene was not re-oxidized at positive holes. Adsorption of nitrobenzene and aniline on TiO_2_ at 25 °C in the presence of 2000 ppm of glucose was investigated. The adsorbed amount of nitrobenzene on TiO_2_ was 2.6 μmol, while aniline adsorption was 0.7 μmol. This result indicates that the priority adsorption of nitrobenzene contributes to effective photocatalytic hydrogenation of nitrobenzene.

**Fig. 2:**
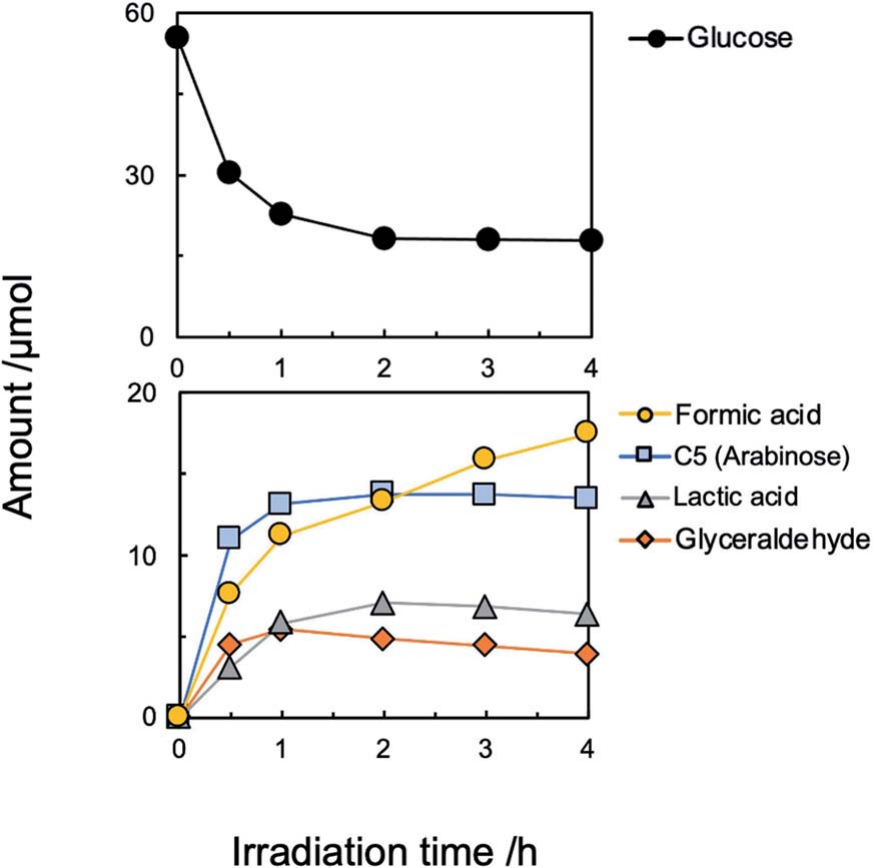
Time courses of the amounts of glucose and oxidation products of glucose in photocatalytic hydrogenation of nitrobenzene (50 μmol) to aniline in the presence of glucose (2000 ppm, 56 μmol) under an Ar condition.

**Scheme 2:**
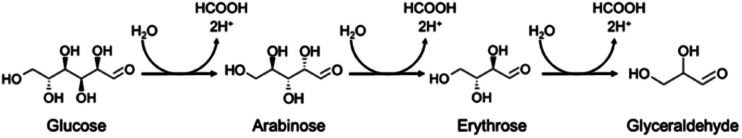
Photocatalytic decomposition of glucose.

Although the amount of adsorption of aniline and nitrobenzene was not so large, the yield of aniline was 81% at 100% conversion of nitrobenzene (Fig. 1). Therefore, strong adsorption of an intermediate(s) and/or side reaction(s) occurred. Hydrogenation of nitrobenzene to aniline involves two kinds of intermediate, *i.e.*, nitrosobenzene and phenyl hydroxyl amine (Scheme 1). However, these intermediates were not detected, and aniline was not increased by further photoirradiation, indicating that a side reaction between nitrobenzene (or intermediate) and glucose (or decomposed compound) proceeded. Although there are some reports of photocatalytic reductions using glucose as a hole scavenger, the product(s) formed from glucose is not discussed in them.^10^Fig. 2 shows time courses of the amounts of oxidized products in photocatalytic hydrogenation of nitrobenzene in the presence of glucose, shown in Fig. 1. Formic acid, C5 compounds (probably arabinose), glyceraldehyde and lactic acid were obtained, which are products that are consistent with the product of the photocatalytic oxidation of glucose (Scheme 2).^10^

Since formic acid formed by decomposition of glucose is an effective hole scavenger in photocatalytic reduction (hydrogenation) of nitro aromatics to amino aromatics,^19^ glucose works as hydrogen source (hole scavenger) in the present system.

A durability test of a photocatalyst is important for its practical applications of photocatalyst. To evaluate the photocatalytic performance of TiO_2_ after the photocatalytic reaction, TiO_2_ powder was recovered from the reaction mixture with filter paper and washed with distilled water and then used repeatedly for the same reaction (Fig. S5†). In second and third cycle, summation of nitrobenzene and aniline was decreased. Photocatalytic activity slightly decreased when TiO_2_ was reused. In third cycle, yield of aniline was decreased 4 μmol of nitrobenzene remained. On the other hand, XRD patterns of TiO_2_ was not changed after reusing (Fig. S6†). These results indicate that oxidation product(s) of glucose was adsorbed on TiO_2_ surface.

## Conclusions

In summary, we succeeded in photocatalytic hydrogenation of nitrobenzene to aniline over bare TiO_2_ using various saccharides without any additives, metal or H_2_ gas. This is the first report of photocatalytic hydrogenation using 10 kinds of saccharides and will be helpful many researchers studying photocatalytic conversion of biomass.

The results obtained in this study satisfy three important requisites in material transformation, *i.e.*, (1) design of a photocatalytic system based on an element strategy that uses abundant elements (without a noble metal or expensive hole scavenger), (2) environmental friendliness that uses no harmful chemicals, and (3) no use of H_2_ gas.

## Author contributions

K. Imamura contributed to conceptualization, funding acquisition, methodology, project administration, supervision and writing. K. Ikeuchi, Y. Sakamoto and Y. Aono and T. Oto contributed to investigation and validation. A. Onda contributed to methodology and writing (review).

## Conflicts of interest

There are no conflicts to declare.

## Supplementary Material

RA-011-D1RA05953J-s001
