# Supplementary material for: Photocatalytic hydrogenation of nitrobenzene to aniline over titanium(iv) oxide using various saccharides instead of hydrogen gas
Source: RSC Adv. 2021 Sep 30;11(51):32300–4. doi: 10.1039/d1ra05953j (PMC9041903; doi:10.1039/d1ra05953j)
Supplement: RA-011-D1RA05953J-s001 [file RA-011-D1RA05953J-s001.pdf]

Electronic Supplementary Material (ESI) for RSC Advances.
This journal is © The Royal Society of Chemistry 2021

Photocatalytic hydrogenation of nitrobenzene to aniline over titanium(IV) oxide using various saccharides instead of hydrogen gas

Kazuya Imamura,*[a] Kazuma Ikeuchi,[a] Yuki Sakamoto,[a] Yushiro Aono,[a] Oto Takahiro[a] and Ayumu Onda[a]

[a] *Research Laboratory of Hydrothermal Chemistry, Faculty of Science and Technology, Kochi University, 2-5-1 Akobonocho, Kochi 780-8073, Japan*

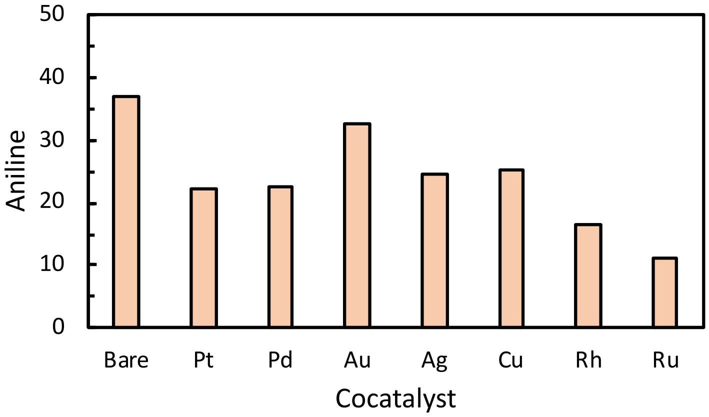

Fig. S1 Effects of different kinds of metal on yield of anilin after 60-min photoirradiation in photocatalytic hydrogenation of nitrobenzene (50 μmol) with glucose (1,000 ppm, 28 μmol) to aniline.

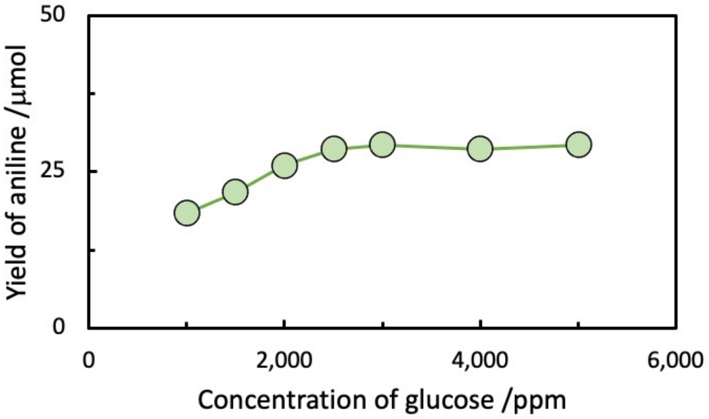

Fig. S2 Effects of different concentrations of glucose on yield of aniline in photocatalytic hydrogenation of nitrobenzene (50 μmol) after 90-min photoirradiation.

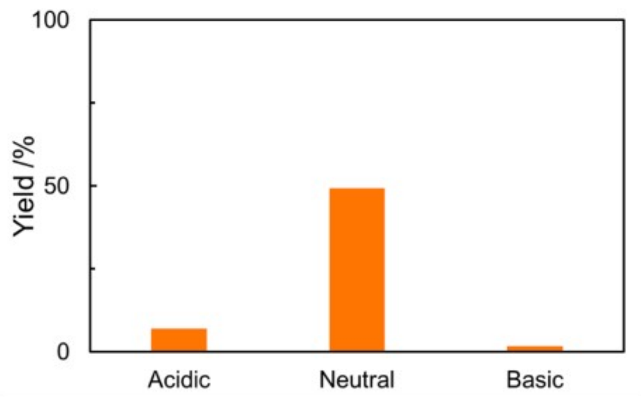

Fig. S3 Effects of different pH values of solution (0.1 mol dm-3 HCl, 0.1 mol dm-3 NaOH) containing glucose (2,000 ppm, 56 μmol) on yield of aniline in photocatalytic hydrogenation of nitrobenzene (50 μmol) after 60 min irradiation.

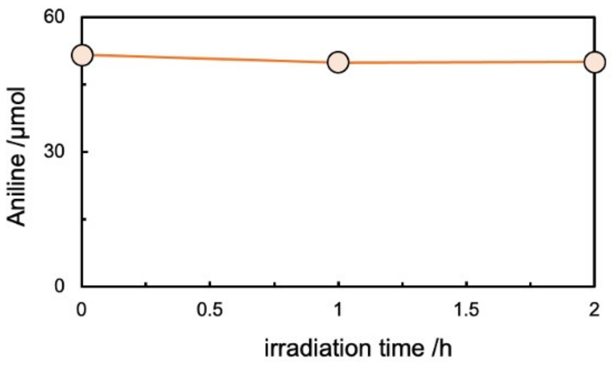

Fig. S4 Photocatalytic reaction of aniline over TiO_2 in the presence of glucose (2,000 ppm) under an Ar condition.

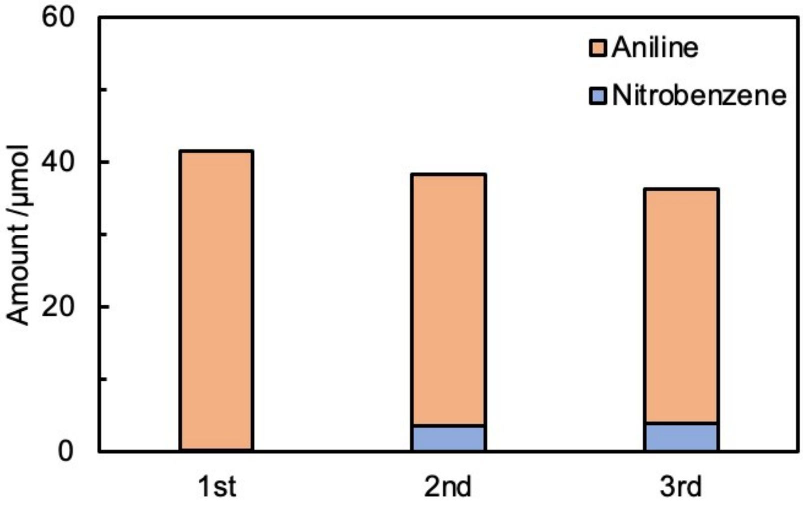

Fig. S5 Durability test of bare TiO_2 in photocatalytic hydrogenation of nitrobenzene to aniline in a aqueous suspension of bare TiO_2 for 2-h photoirradiation.

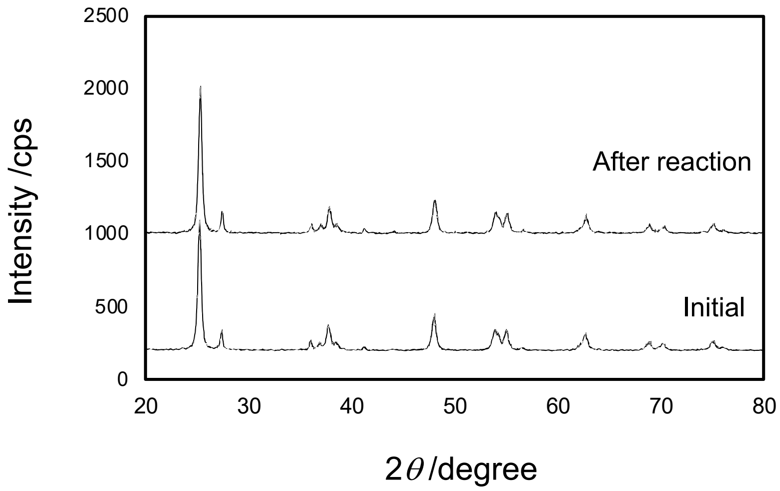

Fig. S6 X-ray diffraction patterns of bare TiO_2 (initial) and after the reaction.
